# Switching to smokeless tobacco as a smoking cessation method: evidence from the 2000 National Health Interview Survey

**DOI:** 10.1186/1477-7517-5-18

**Published:** 2008-05-23

**Authors:** Brad Rodu, Carl V Phillips

**Affiliations:** 1 Department of Medicine, School of Medicine, University of Louisville, Louisville, KY, USA; 2 Department of Public Health Sciences, School of Public Health, University of Alberta, Edmonton, AB, Canada

## Abstract

**Background:**

Although smokeless tobacco (ST) use has played a major role in the low smoking prevalence among Swedish men, there is little information at the population level about ST as a smoking cessation aid in the U.S.

**Methods:**

We used the 2000 National Health Interview Survey to derive population estimates for the number of smokers who had tried twelve methods in their most recent quit attempt, and for the numbers and proportions who were former or current smokers at the time of the survey.

**Results:**

An estimated 359,000 men switched to smokeless tobacco in their most recent quit attempt. This method had the highest proportion of successes among those attempting it (73%), representing 261,000 successful quitters (switchers). In comparison, the nicotine patch was used by an estimated 2.9 million men in their most recent quit attempt, and almost one million (35%) were former smokers at the time of the survey. Of the 964,000 men using nicotine gum, about 323,000 (34%) became former smokers. Of the 98,000 men who used the nicotine inhaler, 27,000 quit successfully (28%). None of the estimated 14,000 men who tried the nicotine nasal spray became former smokers.

Forty-two percent of switchers also reported quitting smoking all at once, which was higher than among former smokers who used medications (8–19%). Although 40% of switchers quit smoking less than 5 years before the survey, 21% quit over 20 years earlier. Forty-six percent of switchers were current ST users at the time of the survey.

**Conclusion:**

Switching to ST compares very favorably with pharmaceutical nicotine as a quit-smoking aid among American men, despite the fact that few smokers know that the switch provides almost all of the health benefits of complete tobacco abstinence. The results of this study show that tobacco harm reduction is a viable cessation option for American smokers.

## Background

For the past half century men in Sweden have had among the lowest rates of smoking – and the lowest rates of smoking-related illnesses – in the developed world [1]. Several recent studies have shown that the high prevalence of smokeless tobacco (ST) use among Swedish men has played a substantial role in the remarkably low smoking prevalence, mainly in two ways. First, the popularity of ST among Swedish men suppresses smoking initiation [2-4]. More importantly, substituting ST facilitates risk reduction by allowing smokers to become smoke-free without abstaining from tobacco and nicotine altogether [3-6], but complete abstinence is still achievable [4,7]. There is now evidence that ST use has started to become popular among Swedish women as well, with similar effects on smoking rates [4,8]. Tobacco harm reduction, which actively encourages inveterate smokers to switch to safer sources of nicotine including ST, is increasingly seen as a promising public health intervention [9-11].

Like Sweden, the U.S. is one of the few Western countries with measurable ST use. According to the National Health Interview Survey (NHIS), the prevalence of ST use among men in the U.S. was 4.5% in the year 2000 [12]. However, in contrast to Sweden, there are only anecdotal reports of ST use for smoking cessation in the U.S [13]. In fact, few resources provide information about cessation at the population level, especially with respect to ST use.

One recent article briefly mentioned that the 2000 NHIS collected information on ST use as a quit-smoking method [14]. However, the information in that article was very selective (1.2% of male former smokers age 36–47 years had switched to snuff or chewing tobacco in order to quit smoking), and it provided little perspective on how switching to ST compared with other cessation methods.

In fact, the 2000 NHIS collected information on 12 methods used by smokers in their most recent quit attempt and who subsequently either quit smoking successfully (former smokers at the time of the survey) or had failed to quit (current smokers). This study uses that survey to estimate the number of male smokers in the U.S. that used various cessation methods.

## Methods

We obtained the 2000 NHIS Adult Sample and Cancer Control Module data files from the Inter-University Consortium for Political and Social Research [15]. Our study focused mainly on men, because in 2000 the prevalence of ST use among women was too low (0.3%)[12] to provide reliable information. However, we generated point estimates of switching to ST among women for comparison.

Subjects who had smoked ≥ 100 cigarettes in their lifetime and who smoked every day or some days were classified as current smokers, while subjects who had smoked ≥ 100 cigarettes in their lifetime and who did not currently smoke were classified as former smokers [16]. Subjects who had used chewing tobacco or snuff 20 times in their life and who used either tobacco product every day or some days were classified as current smokeless tobacco users, while subjects who had used either product 20 times in their life and who did not currently use ST were classified as former users [12]. The cancer control module also asked subjects if they had ever used chewing tobacco or snuff.

In the cancer control module, 3,622 male current smokers were asked: "Have you ever stopped smoking for one day or longer because you were trying to quit smoking?" Those answering "no" (n = 1,325, 37%) were excluded from further analysis regarding cessation attempts. The remaining 2,297 smokers were asked: "The last time you stopped smoking, which of these methods did you use?" Subjects were prompted to "mark all [of the following methods] that apply": (1) stopped all at once (cold turkey), (2) gradually decreased the number of cigarettes smoked in a day, (3) instructions in a pamphlet or book, (4) one-on-one counseling, (5) stop-smoking clinic or program, (6) nicotine patch, (7) nicotine containing gum (such as Nicorette), (8) nicotine nasal spray, (9) nicotine inhaler, (10) Zyban/Bupropion/Wellbutrin medication (abbreviated bupropion here), (11) switched to chewing tobacco or snuff (ST here), and (12) any other method. Information about methods was obtained from 2,180 (95%) of the current smokers who had ever tried to quit. In similar fashion, 3,653 former smokers were asked: "When you stopped smoking completely, which of these methods did you use?" followed by the same choices. Information about methods was obtained from 3,548 former smokers (98%).

We identified the quit methods that are endorsed in the Clinical Practice Guideline (CPG) from the Public Health Service, U.S. Department of Health and Human Services [17]. The survey asked former smokers how long ago they had quit, and we classified these subjects into four groups based on the number of years since quitting: 0–4, 5–14, 15–19 and 20+. Because subjects could select more than one method, the results reported here are not mutually exclusive.

The 2000 NHIS employed a complex design involving stratification, clustering and multistage sampling. We used SPSS statistical software with Complex Samples (Version 15.0 for Windows) to provide estimates, based on the non-institutionalized civilian population of the U.S, of the quit-smoking methods used by the 24.0 million men who had successfully quit smoking (former smokers), and by the 15.1 million men who had attempted to quit but were unsuccessful on their last attempt (current smokers).

## Results

Table 1 provides the number of male survey respondents who had used various methods in their most recent quit attempt and the percentages who were former and current smokers at the time of the survey. An estimated 33 million men reported stopping all at once in their most recent quit attempt; almost 21 million (64%) were former smokers at the time of the survey. Of the 2.9 million men who tried to gradually decrease the number of cigarettes that they smoked, 1.3 million (45%) had become former smokers. Of the 76,000 men following instructions in a pamphlet or book, 28% (21,000) became former smokers.

**Table 1:** Number of male smokers who had tried various methods in their last quit attempt, and the proportions (%) who were former and current smokers at the time of the survey, NHIS 2000

Method	Survey Count^	U.S. Population Estimate^*	% Former (95% CI)	% Current (95% CI)
Stopped all at once	4,822	32,589,195	64 (63–66)	36 (34–37)
Gradually decreased cigarettes smoked	426	2,888,019	45 (40–51)	55 (49–61)
Switched to ST	43	358,668	73 (55–86)	27 (14–45)
Pamphlet/book	11	75,522	28 (9–61)	72 (39–91)
**CPG Endorsed**				
Nicotine patch	393	2,881,084	35 (29–40)	65 (60–71)
Bupropion	138	1,059,982	29 (21–38)	71 (62–79)
Nicotine gum	129	963,692	34 (25–44)	66 (56–75)
Clinic/program	42	310,938	50 (33–67)	50 (33–67)
One-on-one counseling	19	106,501	43 (23–64)	57 (36–77)
Nicotine inhaler	13	98,124	28 (9–61)	72 (39–91)
Nicotine nasal spray	3	14,463	0 (0–35)^+^	100 (65–100)^+^

Any other method	182	1,295,707	63 (54–71)	37 (29–46)

^ Column total exceeds the number of current and former smokers because subjects chose multiple methods.

* Population estimates are reported to the last digit to aid in re-analysis of results. They are not intended to imply a level of precision beyond what can be achieved from the survey.

^+ ^CI is an approximation based on the unweighted survey count.

CI – confidence interval.

ST – smokeless tobacco.

CPG – Clinical Practice Guideline, Department of Health and Human Services.

An estimated 359,000 men switched to ST in their most recent quit attempt, and 73% of them (261,000) were former smokers. In comparison, only 42,000 women switched to ST in their most recent quit attempt, and only 38% of them (16,000) were former smokers at the time of the survey.

Among CPG-endorsed methods, the nicotine patch was used by the largest number of men (estimate, 2.9 million) in their most recent quit attempt, and almost 1 million (35%) were former smokers at the time of the survey. An estimated 1.1 million men used bupropion, and 308,000 (29%) were former smokers. Of the 964,000 men using nicotine gum in their most recent quit attempt, about 323,000 (34%) became former smokers. A stop-smoking clinic/program was used by an estimated 311,000 men, 50% of whom (155,000) became former smokers, the highest proportion among CPG-endorsed methods. Of the estimated 107,000 men who used one-on-one counseling, 45,000 became former smokers (43%). Of the 98,000 men who used the nicotine inhaler in their most recent quit attempt, 27,000 quit successfully (28%). None of the estimated 14,000 men who used the nicotine nasal spray became former smokers. An estimated 1.3 million men used other, unspecified methods in their most recent quit attempt, and 817,000 (63%) became former smokers.

We conducted additional analyses restricted to male former smokers who had quit by using the nicotine patch, nicotine gum, bupropion or by switching to ST (hereafter referred to as switchers), in order to provide a better comparison of these methods. For clarity, we use actual survey numbers and unweighted proportions when reporting these findings. Table 2 provides more information about the use of multiple methods by former smokers who quit by using the three medications or ST. Exclusive use of a single method was more common among patch (70%) and bupropion (64%) users than among gum users or switchers (55%). Forty-two percent of switchers also reported stopping all at once, which was higher than for bupropion (8%), nicotine patch (18%) or nicotine gum (19%). Fifteen percent of switchers reported gradually decreasing the number smoked, which was somewhat higher than for bupropion (3%) or the patch (4%). Multiple medication use was more frequent in former smokers who used gum (26%) or bupropion (21%), compared with former smokers who used the patch (10%).

**Table 2:** Male former smokers who used medications or switched to ST, and their distribution (%) according to other methods used.

Method	Nicotine Patch (n = 128)	Nicotine Gum (n = 42)	Bupropion (n = 39)	Switched to ST (n = 33)
Stopped all at once	18%	19%	8%	42%
Gradually decreased cigarettes smoked	4	10	3	15
Switched to ST	1	5	0	55*
Pamphlet/book	2	5	0	3
Nicotine patch	70*	19	13	3
Bupropion	4	7	64*	0
Nicotine gum	6	55*	8	6
Clinic/program	2	0	0	0
One-on-one counseling	0	0	3	0
Nicotine inhaler	2	2	0	0
Nicotine nasal spray	0	0	0	0
Any other method	1	5	10	3

* Percentage of subjects using only that method.

n – unweighted survey count.

ST – smokeless tobacco.

Note: Column percentages total over 100% because some subjects used multiple methods.

Table 3 shows the distribution of former smokers who used medications or switched to ST, according to the number of years since quitting. Ninety-five percent of bupropion users quit from 0 to 4 years before the survey, while 87% of patch users quit up to 9 years prior to the survey. Although 47% of gum users quit 0–4 years before the survey, the remainder were distributed across the other timeframes, including 20+ years. This pattern was even more evident for switchers, 21% of whom had become former smokers 20+ years prior to the survey.

**Table 3:** Male former smokers who used medications or switched to ST, and their distribution (%) according to the number of years since quitting.

Years Since Quitting	Nicotine Patch (n = 128)	Nicotine Gum (n = 42)	Bupropion (n = 39)	Switched to ST (n = 33)
0–4	60%	47%	95%	40%
5–9	27	14	0	12
10–14	11	17	0	18
15–19	1	17	0	9
20+	1	5	5	21

n – unweighted survey count.

ST – smokeless tobacco

Because separate sets of survey questions were devoted to smoking cessation and smokeless tobacco use, we were able to obtain information about the latter on the 33 switchers. Fifteen of them (46%) were current ST users at the time of the survey, and twelve (36%) were former users. Of the six that were classified as never users, 3 answered yes to the question about ever use of chewing tobacco or snuff.

## Discussion

Anecdotal reports have shown that individual smokers have quit smoking by switching to ST [13]. However, this study provides evidence from a nationally representative survey that switching to ST is a viable, although infrequently attempted, quit smoking method for men in the U.S. Of the 261,000 men who switched to ST and became former smokers, about 120,000 (46%) were current ST users at the time of the survey, indicating that the switch may be permanent for some. On the other hand, 54% of switchers did not use any tobacco product at the time of the survey, suggesting that switching to ST is not incompatible with a goal of achieving complete nicotine and tobacco abstinence.

This study shows that switching to ST resulted in over twice the proportion of former smokers (73%) than the nicotine patch (35%), gum (34%), inhaler (28%) or nasal spray (0%). It is important to note that these percentages do not mean that switching to ST is successful 73% of the time or that using pharmaceutical products have a 30% success rate. This type of study cannot answer the question "How often does a particular method work when tried by a particular individual?" The percentages reported for various methods in our study may be substantially different from corresponding answers to this question. The main reason for the distinction is that the NHIS only collected information about the *most recent *method used. It has no information on the methods used in previous failed quit attempts, or how many times each method was tried.

Regardless of how one interprets the proportions of former and current smokers, it is particularly striking that an estimated 359,000 smokers tried to stop smoking by switching to ST – and over a quarter of a million became former smokers – especially since Americans are largely misinformed about the health risks of ST use [1,18]. For example, in 2005 a survey of 2,028 adult U.S. smokers found that only 11% correctly believed that ST products are less hazardous than cigarettes [19]. In another survey, 82% of U.S. smokers incorrectly believed that chewing tobacco is just as likely to cause cancer as smoking cigarettes [20]. These findings are in direct contrast to the general agreement among tobacco research and policy experts that ST use is far less hazardous than smoking. Although estimates are not precise, ST use likely confers only 0.1% to 10% of the risks of smoking [21-23].

It is safe to assume that rates of switching would increase substantially if smokers knew that switching to ST achieves almost all of the health benefits as quitting tobacco and nicotine altogether [1]. In 2000 the most likely beneficiaries of this knowledge would have been the 1.1 million American men who were dual users of both cigarettes and ST products. These men were already comfortable consuming nicotine from both combusted and smoke-free tobacco. With the knowledge that ST products were 100 times less hazardous than cigarettes, it is conceivable that most would have chosen exclusive use of ST, resulting in a decline of 1.2 percentage points in national adult male smoking prevalence.

Comparison of ST and pharmaceutical nicotine in a regulatory, legal and social context further suggests that the potential of ST as a cessation aid has been under-realized. Nicotine gum and the nicotine patch have been available since 1984 and 1992 respectively [24], and both achieved non-prescription status in 1996, when the manufacturer conducted a large promotional campaign in conjunction with the American Cancer Society Great American Smokeout [25]. In 1999 an estimated $200 million was spent on print and broadcast advertising for smoking cessation products [26].

In contrast to the heavy promotion and advertising of pharmaceutical nicotine products for smoking cessation in the late 1990s, the environment for ST products was quite negative. A ban on broadcast advertising of ST had been established as early as 1986 [27], so the estimated $170 million spent by manufacturers in 1999 was restricted largely to print media and other forms of advertising and promotion [28]. Not only were manufacturers effectively prohibited from offering ST products as reduced-risk options for smokers, a counter-marketing program was launched by congressional legislation in 1986, in the form of a mandatory warning on every third package of ST sold in the U.S.: "This product is not a safe alternative to cigarettes" [27]. In addition, major efforts have been made by the American tobacco control community to impede any widespread transition from cigarettes to ST [1,18]. Despite the pro-pharmaceutical and anti-ST climate, an estimated 261,000 men had used smokeless tobacco to quit smoking by the year 2000. While this number is lower than the number who had successfully used the nicotine patch (about one million), it is comparable to the number who had successfully used either nicotine gum or antidepressants, and far more than the number who were successful with other pharmaceutical nicotine products.

We expected to find evidence in later surveys that increasing awareness of the low risk profile of modern, socially acceptable ST products would have resulted in heightened popularity for this cessation method. Unfortunately, no information on switching to ST is available in subsequent NHIS surveys, because that option was removed when the Cancer Control module appeared again in the 2005 NHIS [29]. It is possible that individuals responsible for designing the module expected an increase in switching as well, and that they chose to not find out.

A major strength of this study is that it is based on the survey series that the Centers for Disease Control and Prevention (CDC) uses for national smoking prevalence estimates [16]. In fact, our findings were produced from the very same dataset (and specific survey questions) used by the American Cancer Society in a recent study of smoking cessation treatments used by American smokers [30]. Thus, we were surprised when a senior Cancer Society scientist, who was a coauthor on that study [30], stated emphatically that "There is no evidence that smokers will switch to ST products and give up smoking" [31]. Although the Cancer Society has not endorsed tobacco harm reduction, its scientists certainly know that there is unequivocal evidence from the 2000 NHIS survey that 261,000 smokers have switched to ST products in order to quit smoking.

Studies based on survey data are limited by the nature of the survey instrument and the quality of self-reported information. With respect to this survey, current and former smokers were encouraged to choose multiple methods that were not mutually exclusive, which creates some difficulty in reporting the results and may be confusing for some readers. For example, "Stopped all at once (cold turkey)" was so frequently chosen (with or without other methods) – as would be expected – that all other methods pale in direct comparison. That comparison is certainly confusing, but it may also be inappropriate, since the cold turkey response is orthogonal to the other methods. However, excluding this item would have eliminated information that some readers consider useful. Our goal was to present a complete picture of the data, including how frequently all of the methods were chosen.

We noted some inconsistencies among former smokers using medications and switching to ST. For example, among the 128 former smokers who used the nicotine patch, 16 reported that they quit before the patch became available. Two subjects using nicotine gum and two using bupropion had similar inconsistencies. In addition, for three subjects who switched to ST, their responses to other questions indicated no ST use. It is not possible to resolve these irregularities in a systematic manner, but they may affect the certainty of the estimates.

## Conclusion

This study documents that switching to ST compares very favorably with pharmaceutical nicotine as a quit-smoking aid among American men, despite the fact that few smokers know that the switch provides almost all of the health benefits of complete tobacco abstinence. As long as American smokers are misinformed about the comparative risks of ST and cigarettes, most will not consider trying to switch, or will do so only reluctantly. A social and public health environment that honestly informs smokers about comparative risks would provide many more smokers with the opportunity to lead longer and healthier lives.

## Competing interests

This study was supported by unrestricted grants from smokeless tobacco manufacturers to the University of Louisville (US Smokeless Tobacco Company and Swedish Match AB) and to the University of Alberta (USSTC). The terms of the grants assure that the grantors are unaware of this study, and thus had no scientific input or other influence with respect to its design, analysis, interpretation or preparation of the manuscript.

Dr. Rodu has no financial or other personal relationship with regard to the grantors. Dr. Phillips has provided consulting services to USSTC in the context of product liability litigation.

## Authors' contributions

Both authors made substantive contributions to all aspects of this study, and both approve the final manuscript.
